# Ticking Bomb: Asymptomatic Mirizzi Syndrome

**DOI:** 10.7759/cureus.1854

**Published:** 2017-11-16

**Authors:** Stella Pak, Damian Valencia, Brendan Sheehy, Uchenna Agbim, Yusuf Askaroglu, Christine Dee

**Affiliations:** 1 Internal Medicine, Kettering Medical Center; 2 Department of Medicine, Kettering Medical Center; 3 Department of Medicine, Boonshoft School of Medicine; 4 Wright State University Boonshoft School of Medicine

**Keywords:** gallstone, mirizzi syndrome, cholecystectomy

## Abstract

Mirizzi syndrome, also known as extrinsic biliary compression syndrome, is a rare clinical entity in which the common bile duct is obstructed by compression by the impaction of one or more gallstones in the cystic duct or gallbladder infundibulum. This case illustrates an absolutely asymptomatic presentation of Mirizzi syndrome in a 62-year-old, otherwise healthy, woman. Mirizzi syndrome was treated with preemptive laparotomy cholecystectomy. The present case is exemplary for careful evaluation with the proper index of suspicion in establishment of preoperative diagnosis as well as prompt treatment prior to development of complications.

## Introduction

Mirizzi syndrome, also known as extrinsic biliary compression syndrome, is a rare clinical entity in which the common bile duct is obstructed by compression by the impaction of one or more gallstones in the cystic duct or gallbladder infundibulum [1].^ ^Mirizzi syndrome usually develops as a chronic complication of symptomatic gallstone disease. The pathophysiology involves an inflammatory reaction where a pressure ulcer is caused by an impacted gallstone at the gallbladder infundibulum, causing obstruction of the bile duct, leading to a host of complications including cholecystocholedochal or cholecystohepatic fistulas [2].^ ^Some common presenting symptoms can be acute, chronic and unspecific; however, presentation could include a constellation of obstructive jaundice, abdominal pain, and fever in a patient with known or suspected gallstone disease [3]. Diagnostic findings on laboratory results include hyperbilirubinemia, elevated level of aminotransaminases, and leukocytosis. Extremely high levels of malignancy markers cancer antigen (CA) 19-9 have been found in patients and often have been mistakenly and incorrectly labelled as malignancy.

Total mortality associated with Mirizzi syndrome is estimated to range from 5% to 31% [1]. Herein, we present a case of asymptomatic presentation of Mirizzi syndrome in a 62-year-old, otherwise healthy, woman. This case illustrates that Mirizzi, which can lead to a cascade of life-threatening complications, can present without any symptoms.

## Case presentation

An otherwise healthy 62-year-old female was found to have abnormal liver function studies in a comprehensive metabolic panel ordered as a part of annual wellness visit (aspartate transaminase 63 U/L, alanine aminotransferase 78 U/L, alkaline phosphatase 301 U/L, total bilirubin 0.5 µmol/L, direct bilirubin 0.3 µmol/L, and indirect bilirubin 0.2 µmol/L). This patient did not have any chronic medical conditions and was taking an over-the-counter multivitamin tablet only. She was absolutely asymptomatic without abdominal pain, jaundice, nausea or vomiting. She denied any episodes of postprandial epigastric pain or intermittent nausea, bloating or belching. She had a blood pressure of 138/84 mmHg, heart rate of 60/min, respiratory rate of 18/min, body temperature of 98.0 °F, and oxygen saturation level of 98% on room air. Her abdomen was soft without any palpable masses, tenderness, rigidity, or guarding. Later, work-up imaging with computerized tomography revealed dilated intrahepatic bile ducts.

**Figure 1:**
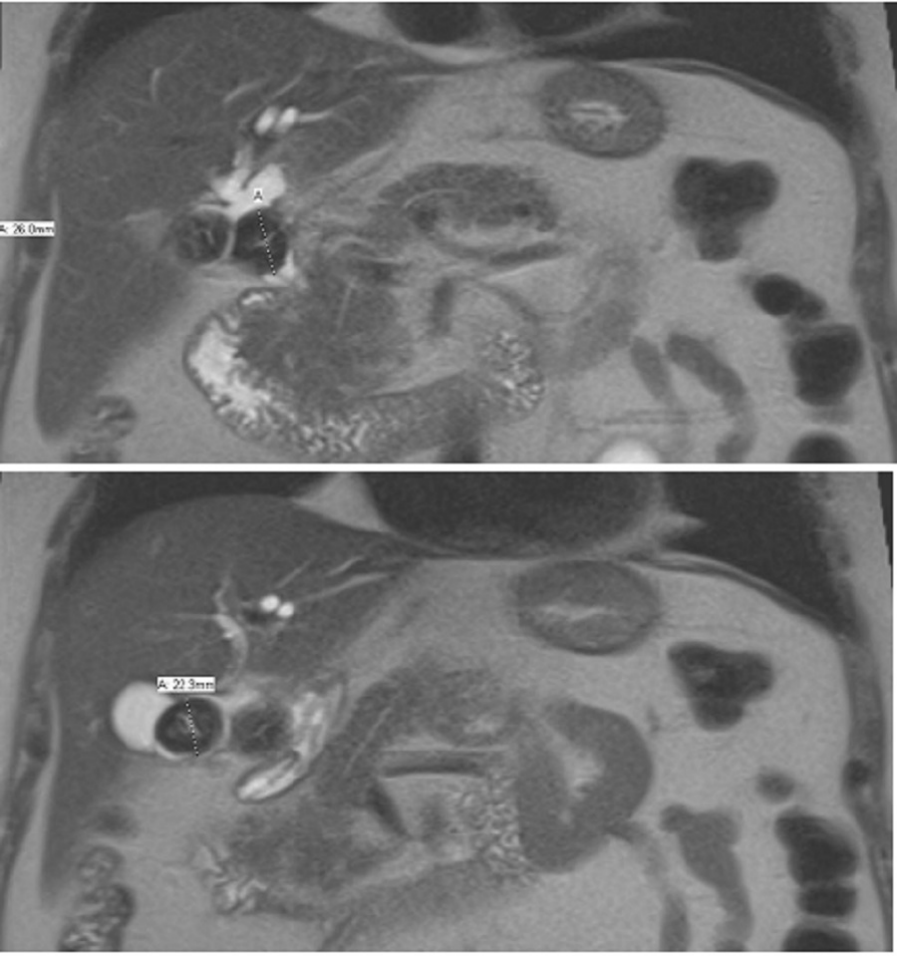
Computerized tomography of the abdomen showing two gallbladder stones The first stone measures 2.9 x 2.2 cm in the neck of the gallbladder (top image), the second stone measures 2.4 x 2.6 cm in the proximal body of the gallbladder (bottom image).

**Figure 2:**
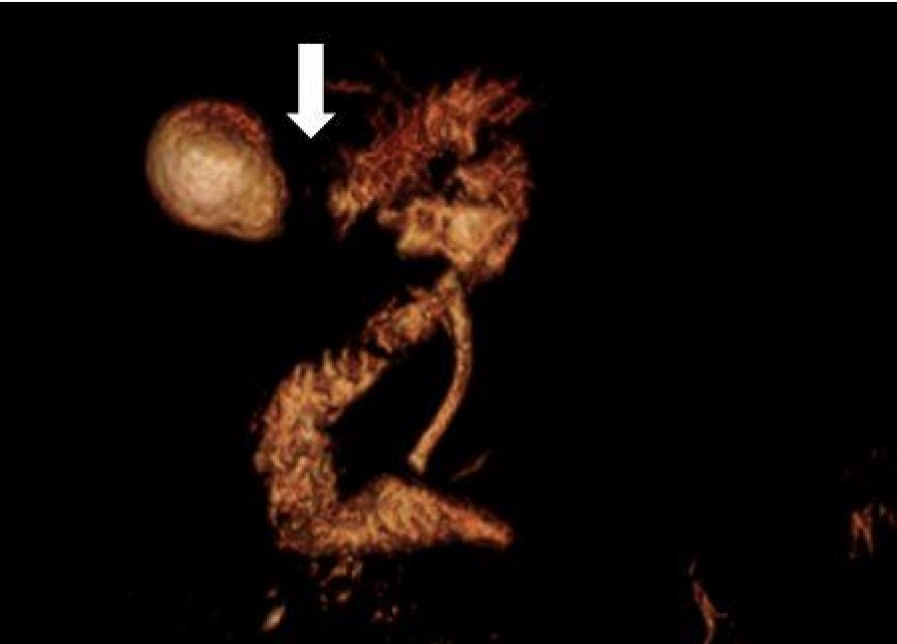
Magnetic resonance cholangiopancreatography with three-dimensional reconstruction demonstrating obstruction in bile duct at the neck and proximal body of gallbladder

**Figure 3:**
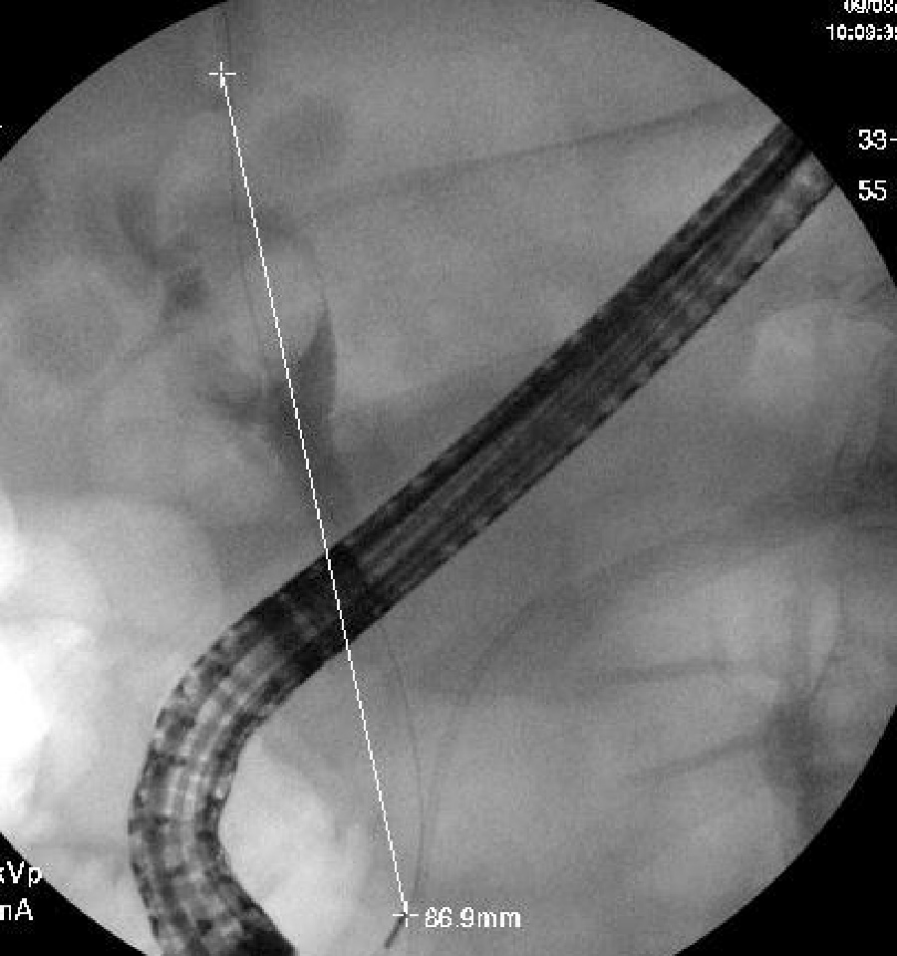
Endoscopic retrograde cholangiopancreatography visualizing biliary stenting, measuring approximately 86.9 mm

These findings prompted further investigation with magnetic resonance cholangiopancreatography (MRCP), which then showed two large lamellated gallstones within the neck and proximal body of the gallbladder (Figure 1). The large stone in the neck of the gallbladder was compressing the intrahepatic biliary duct, consistent with diagnosis of Mirizzi’s syndrome (Figure 2). The wall of the bladder was thickened and irregular, suggesting chronic cholecystitis. Tumor markers, including cancer antigen (CA) 19-9, CA-125, and carcinoembryonic antigen were all negative. Endoscopic retrograde cholangiopancreatography (ERCP) visualized a filling defect at the proximal extrahepatic biliary tree with mild intrahepatic bile duct dilation. Biliary and pancreatic stents were placed via ERCP (Figure 3).

To prevent further complications, including pancreatitis and cholangitis, our patient chose to undergo preemptive laparoscopic cholecystectomy. During the operation, the gallbladder wall was noted to be extremely thickened with chronic inflammatory changes and fibrosis. Furthermore, there was a large gallstone at the dome of the gallbladder, making grasping and retraction of the gallbladder difficult, necessitating conversion to open laparotomy. Within the gallbladder, two brown calculi measuring 2.2 cm and 3.0 cm were seen. Our patient had an uneventful postoperative course and recovery.

## Discussion

Establishing diagnosis of Mirizzi syndrome is challenging due to non-specific symptoms, rarity, and resemblance of its radiographic findings to cholecholithiasis, bile duct stricture, or cholangiocarcinoma. Preoperative establishment of diagnosis can help minimize morbidity and mortality from Mirizzi syndrome by preventing the development of complications, such as cholecystobiliary or cholecystoenteric fistula [4]. Mirizzi syndrome should be considered, when history and physical are suspicious, for biliary pathologies such as right upper quadrant pain, jaundice, and fever. Suspicions should be confirmed with subsequent ultrasound or abdominal CT. Ultrasound imaging has a sensitivity of 23-46%, and abdominal CT is similarly limited in regard to differentiating Mirizzi syndrome from other biliary pathologies [5]. Ultimately, direct cholangiography should be performed in all suspected cases. MRCP T2-weighted imaging has a sensitivity of 93% for recognizing calculi and possible malignancy within the pancreaticobiliary system [6]. ERCP is also capable of confirming the diagnosis of Mirizzi syndrome [5]. Other advantages of ERCP include identifying cholecystobiliary fistulae and the ability for biliary decompression with stent placement for relief of obstructive symptoms such as jaundice and abdominal pain [7]. Endoscopic removal of stones also decreases the potential for common bile duct exploration intraoperatively [8]. ERCP may also be the best option for poor surgical candidates.

Gallbladder cancer may present as a complication of Mirizzi syndrome. ERCP and MRCP are both capable of identifying extraluminal signs of malignancy and therefore must be performed when there is suspicion of Mirizzi syndrome. Once Mirizzi syndrome is identified and imaging tests are confirmatory, surgical cholecystectomy is the definitive treatment option and the recommended option for eligible patients. If Mirizzi syndrome is not identified preoperatively but is instead found during surgery, intraoperative cholangiogram must be performed to confirm the diagnosis and explore any abnormalities in biliary anatomy [9-10].

## Conclusions

In the view of possible complications and close association of Mirizzi syndrome with cholangiocarcinoma, our patient underwent preemptive cholecystectomy. The present case is exemplary for careful evaluation with the proper index of suspicion in the establishment of preoperative diagnosis and prompt treatment prior to development of complications.
